# Influence of Electrical Resistivity and Machining Parameters on Electrical Discharge Machining Performance of Engineering Ceramics

**DOI:** 10.1371/journal.pone.0110775

**Published:** 2014-11-03

**Authors:** Renjie Ji, Yonghong Liu, Ruiqiang Diao, Chenchen Xu, Xiaopeng Li, Baoping Cai, Yanzhen Zhang

**Affiliations:** College of Electromechanical Engineering, China University of Petroleum, Qingdao, Shandong, China; Massachusetts Institute Of Technology, United States of America

## Abstract

Engineering ceramics have been widely used in modern industry for their excellent physical and mechanical properties, and they are difficult to machine owing to their high hardness and brittleness. Electrical discharge machining (EDM) is the appropriate process for machining engineering ceramics provided they are electrically conducting. However, the electrical resistivity of the popular engineering ceramics is higher, and there has been no research on the relationship between the EDM parameters and the electrical resistivity of the engineering ceramics. This paper investigates the effects of the electrical resistivity and EDM parameters such as tool polarity, pulse interval, and electrode material, on the ZnO/Al_2_O_3_ ceramic's EDM performance, in terms of the material removal rate (MRR), electrode wear ratio (EWR), and surface roughness (SR). The results show that the electrical resistivity and the EDM parameters have the great influence on the EDM performance. The ZnO/Al_2_O_3_ ceramic with the electrical resistivity up to 3410 Ω·cm can be effectively machined by EDM with the copper electrode, the negative tool polarity, and the shorter pulse interval. Under most machining conditions, the MRR increases, and the SR decreases with the decrease of electrical resistivity. Moreover, the tool polarity, and pulse interval affect the EWR, respectively, and the electrical resistivity and electrode material have a combined effect on the EWR. Furthermore, the EDM performance of ZnO/Al_2_O_3_ ceramic with the electrical resistivity higher than 687 Ω·cm is obviously different from that with the electrical resistivity lower than 687 Ω·cm, when the electrode material changes. The microstructure character analysis of the machined ZnO/Al_2_O_3_ ceramic surface shows that the ZnO/Al_2_O_3_ ceramic is removed by melting, evaporation and thermal spalling, and the material from the working fluid and the graphite electrode can transfer to the workpiece surface during electrical discharge machining ZnO/Al_2_O_3_ ceramic.

## Introduction

Advances in materials engineering have led to the development of a generation of materials which present better properties for their use in different industrial sectors. Between them, engineering ceramics are widely employed in the manufacturing industry, defense industry, aerospace, and other fields due to their high hardness, high temperature strength and high corrosion resistance [1]. However, their machinability when conventional machining methods are used is not good due to their poor thermal properties, high wear and corrosion resistance, brittleness and hardness properties [2]–[5].

Electrical discharge machining (EDM) is a most popular material removal process based on the concept of material removal by electric discharges. Each of those discharges removes a small amount of material from the workpiece due to the high temperature during the discharge process. The process can machine any material provided that it can conduct electricity, so the process is used to machine very hard conductive materials. Brittle materials such as engineering ceramics can also be machined by this process because that there is no contact between electrode and workpiece leads to the absence of forces during the erosion [6]–[8].

However, the electrical resistivity of the popular engineering ceramics is higher than that of metal, which makes machining engineering ceramics more difficult than machining metal using EDM. Recently, some researches about EDM performance of engineering ceramics with different electrical resistivities have been made.

The unidirectional conductivity of semiconductor crystals with the electrical resistivity of 2.1 Ω·cm during EDM has been investigated by Qiu et al. [9]. The results show that the semiconductor crystals with the electrical resistivity of 2.1 Ω·cm can be directly machined by EDM. W. König et al. [10] have found that the high removal rate as compared with traditional techniques for machining ceramic materials can be obtained provided that their electrical resistivity is below of 100 Ω·cm. Liu and co-workers [11]–[12] have investigated electrical discharge milling SiC ceramics with the electrical resistivity of 500 Ω·cm, and found that the process is able to effectively machine SiC ceramics with the electrical resistivity of 500 Ω·cm due to the good flushing and quickly cooling in the discharge gap. The electrical resistivity versus TiN content in TiN/Si_3_N_4_ composites has been studied by Liu et al. [13]. The results show that the electrical resistivity of the composites is approximately 0.01 Ω·cm when TiN content is higher than 30 vol.%, so that EDM can be used to machine this material. Tak et al. [14] have investigated the characteristic evaluation of aluminum oxide (Al_2_O_3_)/carbon nanotubes (CNTs) hybrid composites with different CNTs contents for micro-electrical discharge machining, and they found that the electrical resistivity has a decreasing tendency as the CNTs content is increased, the electrical resistivity has a critical point at Al_2_O_3_/5% CNTs, and the dimensional accuracy and circularity of Al_2_O_3_/5% CNTs composites are superior to other ones due to homogeneous distribution of CNTs in matrix. Hanaoka and co-workers [15] have fabricated Si_3_N_4_ composites containing carbon nanotube and graphene Nano platelet with different contents. The results show that the lower material removal rate, better electrode wear ratio, and better surface roughness could be obtained on the conductive materials than insulating materials. Moreover, the Si_3_N_4_ composite containing 0.9 vol.% for carbon nanotube with the electrical conductivity of 0.04 S·m^−1^, can be machined by the normal EDM method, but the rougher hole edge shape is obtained when the Si_3_N_4_ composite is machined by the normal EDM method, compared with the assisting electrode EDM method.

It is also known that when the electrical resistivity of the engineering ceramics is low, the electrical discharge of the engineering ceramics is stable, and the machining performance is fine. As the electrical resistivity of the engineering ceramics increases, the discharge instability occurs, which will deteriorate the machining performance. But so far there have been no research papers about the effect of the electrical resistivity on EDM performance by the authors. Moreover, there is yet no studying about the relationship between the EDM parameters and the electrical resistivity of the engineering ceramics when applying the EDM technology. Therefore, the authors have decided to investigate the effects of the electrical resistivity and machining parameters, such as tool polarity, pulse interval, and electrode material, on the process performance in terms of the material removal rate (MRR), electrode wear ratio (EWR), and surface roughness (SR Ra).

## Experimental Procedures

**Figure 1:**
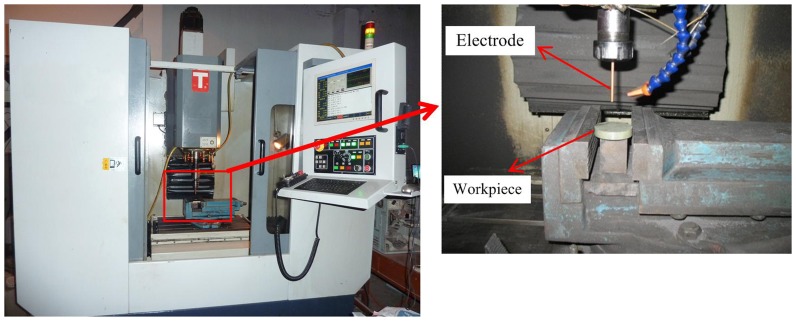
EDM milling machine for machining ZnO/Al_2_O_3_ ceramic.

The experiment was performed in a self-developed EDM milling machine, as illustrated in Figure 1. The workpiece was ZnO/Al_2_O_3_ ceramic with different Al_2_O_3_ contents. The electrical resistivity of the ZnO/Al_2_O_3_ ceramic was measured with a pointer insulation resistance tester (PC40B, Shanghai Anbiao Electronics Corporation, China) and a digital ohmmeter (SD2002, Shanghai Qianfeng Electronic Instrument Corporation, China). The electrical resistivity of the ZnO/Al_2_O_3_ ceramics with different Al_2_O_3_ contents was listed in Table 1.

**Table 1:** The electrical resistivity of ZnO/Al_2_O_3_ ceramic with different Al_2_O_3_ content.

Al_2_O_3_ content (Wt%)	0	0.01	0.05	0.1	0.5	1	1.5	2
Electrical resistivity (Ω·cm)	324000	3410	2470	687	15	11.64	9	6.3

The weighings of the workpiece and the electrode before and after machining were measured by an electron balance (Sartorius BS224S, Germany). The material removal rate (MRR) and electrode wear ratio (EWR) were calculated according to Equations (1) and (2), respectively. The surface roughness (SR) was measured by a surface roughness tester (TR300, Qingdao Times Instrument Corporation, China). The microstructure of the workpiece surface was examined with an electron probe micro-analyzer (EPMA JEOL JXA-8230, Japan), equipped with an energy dispersive spectrometer (EDS Oxford Inca X-Act). All the observed specimens had been cleaned ultrasonically and dried with a hot-air blower before the examination. 
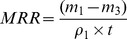
(1)

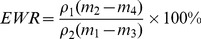
(2)where *m_1_*, *m_2_* were the weighings of workpiece and electrode before machining, respectively, *m_3_*, *m_4_* were the weighings of workpiece and electrode after machining, respectively, *ρ_1_*, *ρ_2_* were the densities of workpiece and electrode, respectively, *t* was the machining time.

Unless otherwise specified, the following experimental parameters were used: The electrode was copper rod 4 mm in diameter and a length of 10 mm, the tool polarity was negative, the pulse duration was 200µs, the pulse interval was 50µs, the open-circuit voltage was 150V, the discharge current was 50A, and the working fluid was composed of 10 mass% emulsified oil and 90 mass% distilled water, which were mixed with a constant speed power-driver mixer.

## Results and Discussion

### Effect of the electrical resistivity on the process performance

**Figure 2:**
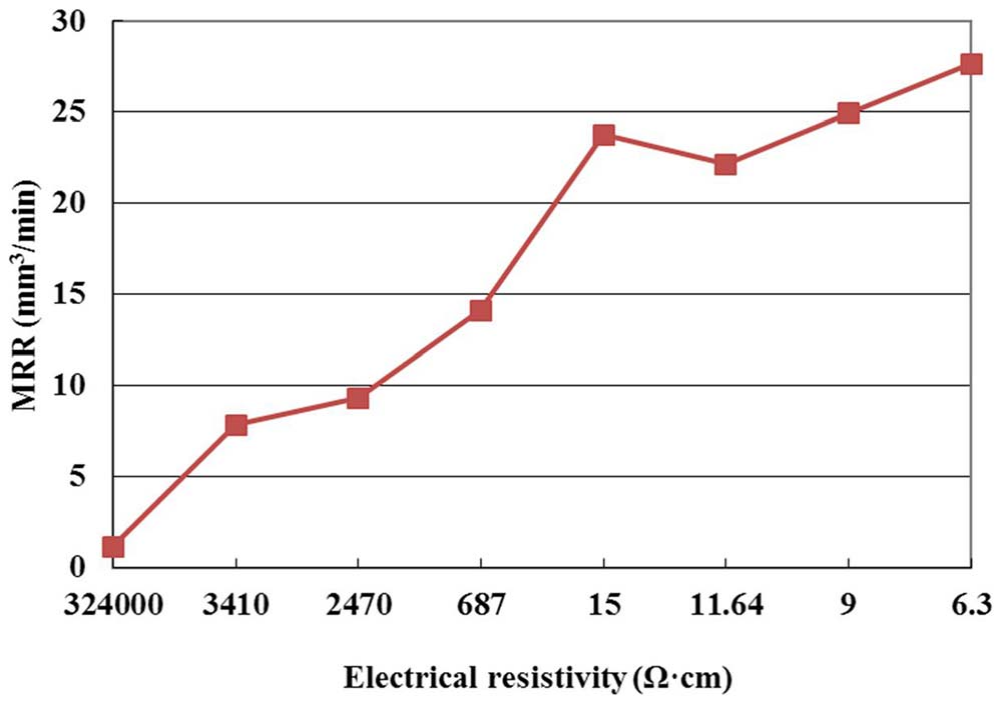
Effect of the electrical resistivity on MRR.

The effect of the electrical resistivity on MRR is illustrated in Figure 2, for the positive tool polarity and pulse interval of 20µs. As shown in this figure, the MRR increases with the decrease of electrical resistivity. This phenomenon can be explained as follows. As the electrical resistivity decreases, the discharge channel is formed easily, the discharge delay time decreases, the discharge becomes violent, and the discharge energy released to the workpiece at the same time increases, so the MRR increases.

**Figure 3:**
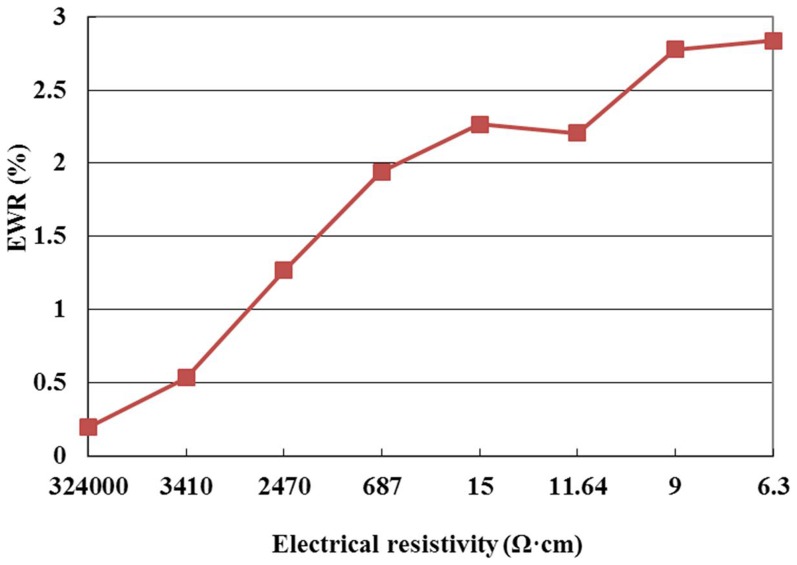
Effect of the electrical resistivity on EWR.

Figure 3 shows the effect of the electrical resistivity on EWR, for the positive tool polarity and pulse interval of 20µs. It can be seen from this figure that the EWR increases with the decrease of electrical resistivity. The reason for this is that when the electrical resistivity decreases, the discharge channel is formed easily, and the electrode material removal increases; therefore the EWR increases.

**Figure 4:**
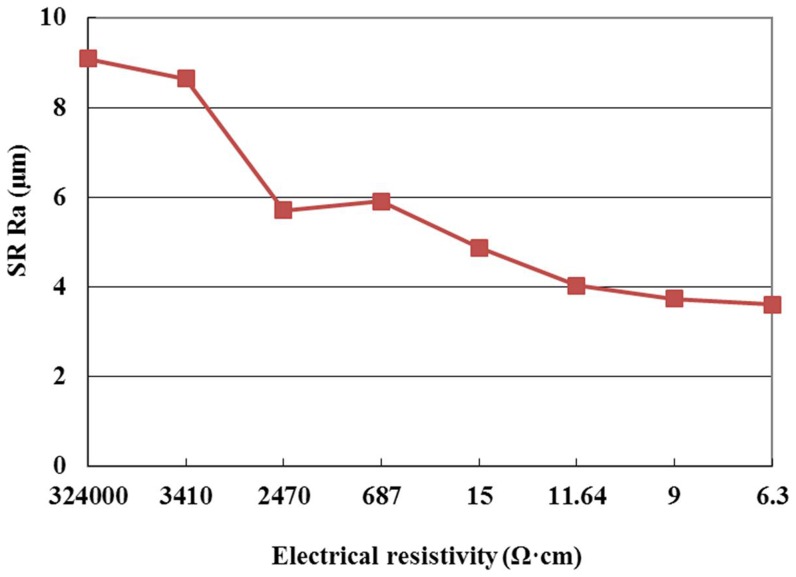
Effect of the electrical resistivity on SR.

SR with positive tool polarity and pulse interval of 20µs is given in Figure 4. The figure indicates that the SR decreases with the decrease of electrical resistivity. Many things can cause this phenomenon. When the electrical resistivity decreases, the discharge occurs easily, and the workpiece is removed by vaporization and melting, which makes the workepiece surface smooth, so the SR decreases with the decrease of electrical resistivity.

### Effect of the tool polarity on the process performance

**Figure 5:**
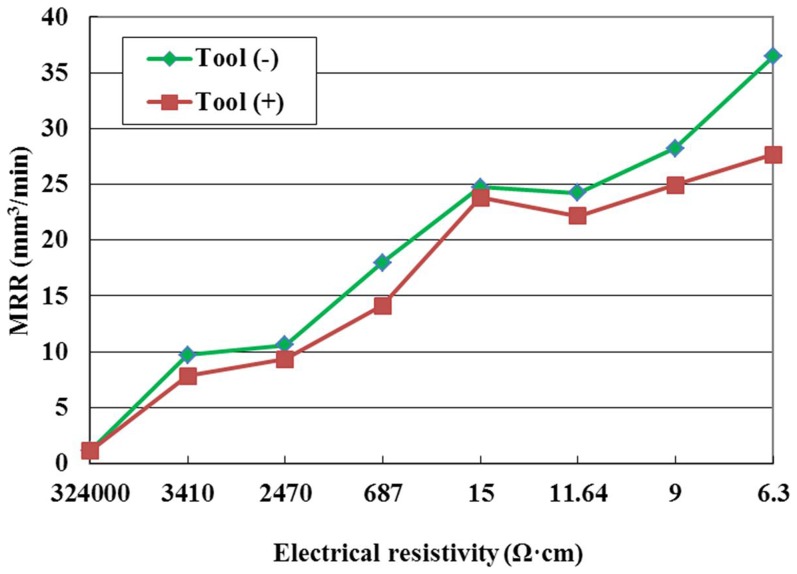
Effect of the tool polarity on MRR.

The effect of the tool polarity on MRR is illustrated in Figure 5 for the pulse interval of 20µs. As shown in this figure, the MRR with positive tool polarity is lower than that with negative tool polarity at the same electrical resistivity. There are many reasons causing this phenomenon. Compared with metal, the electrical resistivity of ZnO/Al_2_O_3_ ceramic is higher, the discharge delay time is longer, and the effective discharge time during a pulse is shorter. Because the mass of the electrons is much smaller than that of positive ions, they can be accelerated quickly during a short time, the bombardment effect by electrons is stronger than that by positive ions; therefore the MRR is high in negative tool polarity.

**Figure 6:**
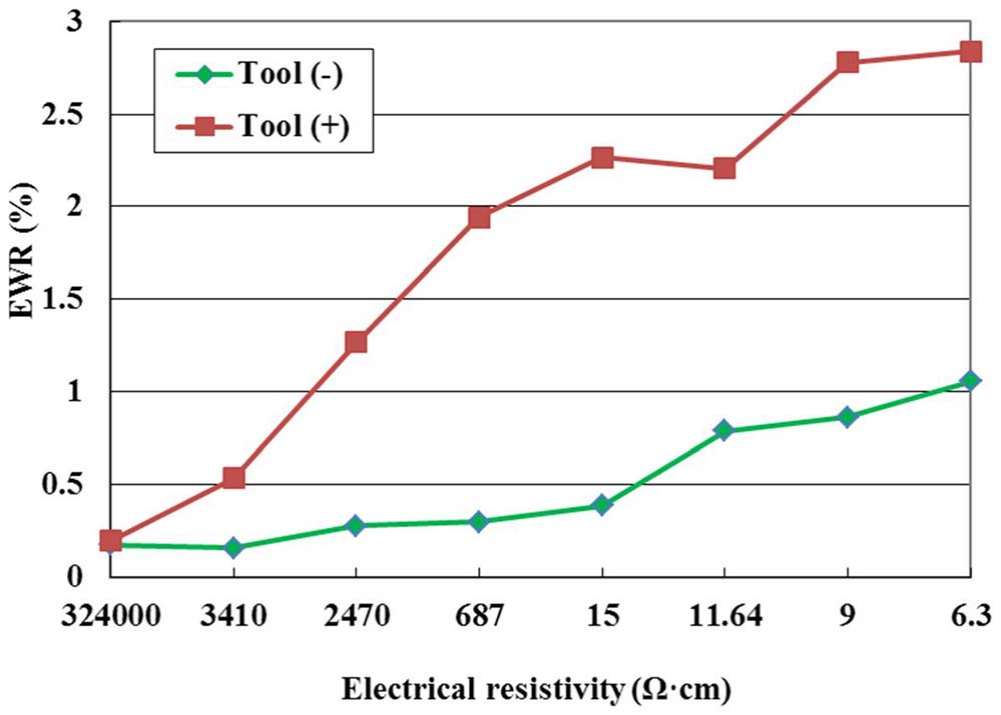
Effect of the tool polarity on EWR.

Figure 6 shows the effect of the tool polarity on EWR for the pulse interval of 20µs. It can be seen from this figure that the EWR with the positive polarity is higher than that with the negative polarity at the same electrical resistivity. As mentioned above, the bombardment effect by the electrons is stronger than that by positive ions during electrical discharge machining ZnO/Al_2_O_3_ ceramic, so the electrode wear ratio is high in positive tool polarity.

**Figure 7:**
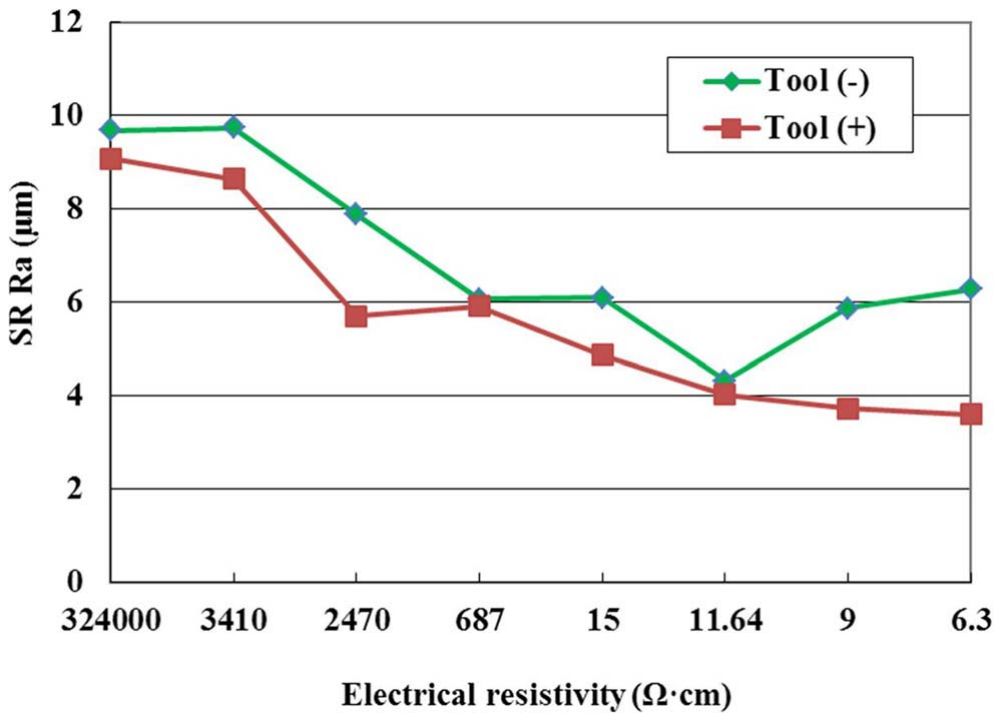
Effect of the tool polarity on SR.

The effect of the tool polarity on SR is shown in Figure 7 for the pulse interval of 20µs. The figure indicates that under the same conditions the SR in negative tool polarity is higher than that in positive tool polarity. This phenomenon can be explained as follows. The bombardment effect by electrons is stronger than that by positive ions, the craters on the workpiece surface produced by electrons are deep; therefore, the SR is high in negative tool polarity. It can also be seen from Figure 7 that the SR increases with the decrease of electrical resistivity with the negative tool polarity when the electrical resistivity is low. The reason for this is that when the electrical resistivity is lower than 11.64 Ω·cm, the discharge channel is easily formed, the bombardment effect by electrons is strong, the discharge crater is large with the negative tool polarity, so the SR increases with the decrease of electrical resistivity.

### Effect of the pulse interval on the process performance

**Figure 8:**
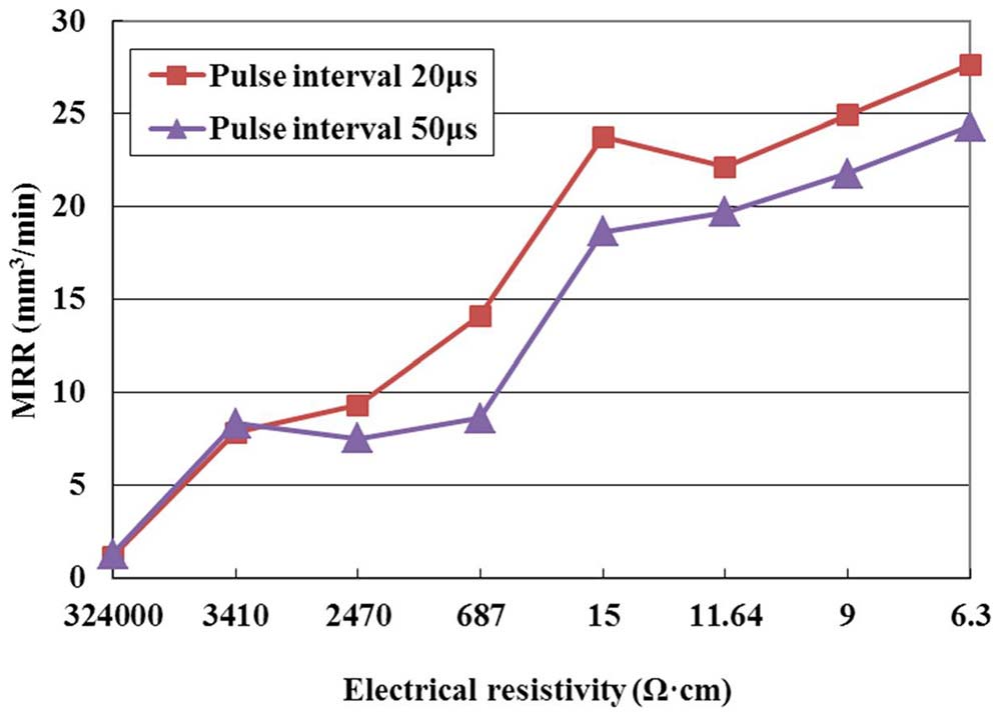
Effect of the pulse interval on MRR.

The effect of the pulse interval on MRR is illustrated in Figure 8 for the positive tool polarity. As shown in this figure, the MRR decreases with the increase of pulse interval at the same electrical resistivity. This phenomenon can be explained as follows. The discharge frequency and the material removal in a unit time decrease with an increase in pulse interval, so the MRR with the pulse interval of 50µs is lower than that with the pulse interval of 20µs at the same electrical resistivity.

**Figure 9:**
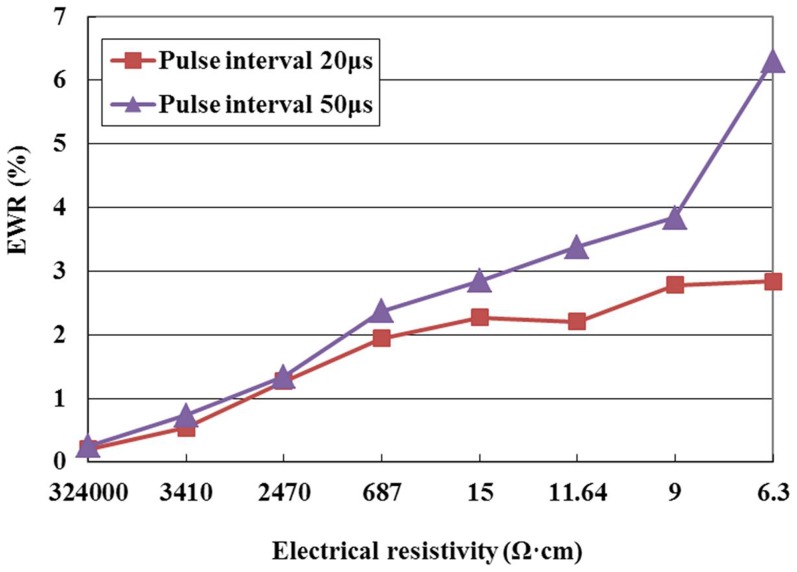
Effect of the pulse interval on EWR.

Figure 9 shows the effect of the pulse interval on EWR for the positive tool polarity. It can be seen from this figure that the EWR increases with the increase of pulse interval at the same electrical resistivity. Many things can cause this phenomenon. As the pulse interval increases, the time for deionization of the dielectric increases, the discharge energy delivered to the machining gap decreases in a unit time, the released carbon decomposed from the dielectric decreases, the deposition effect weakens, and the electrode wear compensation decreases [16]; therefore, the EWR increases.

**Figure 10:**
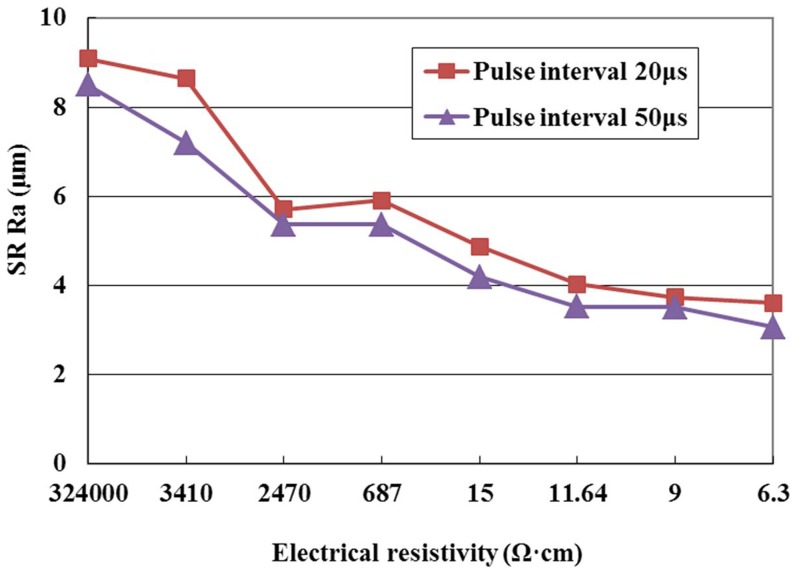
Effect of the pulse interval on SR.

SR with different pulse interval settings is given in Figure 10 for the positive tool polarity. It can be seen from this figure that under the same conditions the SR decreases with the increase of pulse interval. This phenomenon can be explained as follows. A longer pulse interval means more time for deionization of the dielectric, the discharge is stable, and the amount of craters generated by EDM is less; therefore, the SR with the pulse interval of 50µs is lower than that with the pulse interval of 20µs at the same electrical resistivity.

### Effect of the electrode material on the process performance

**Figure 11:**
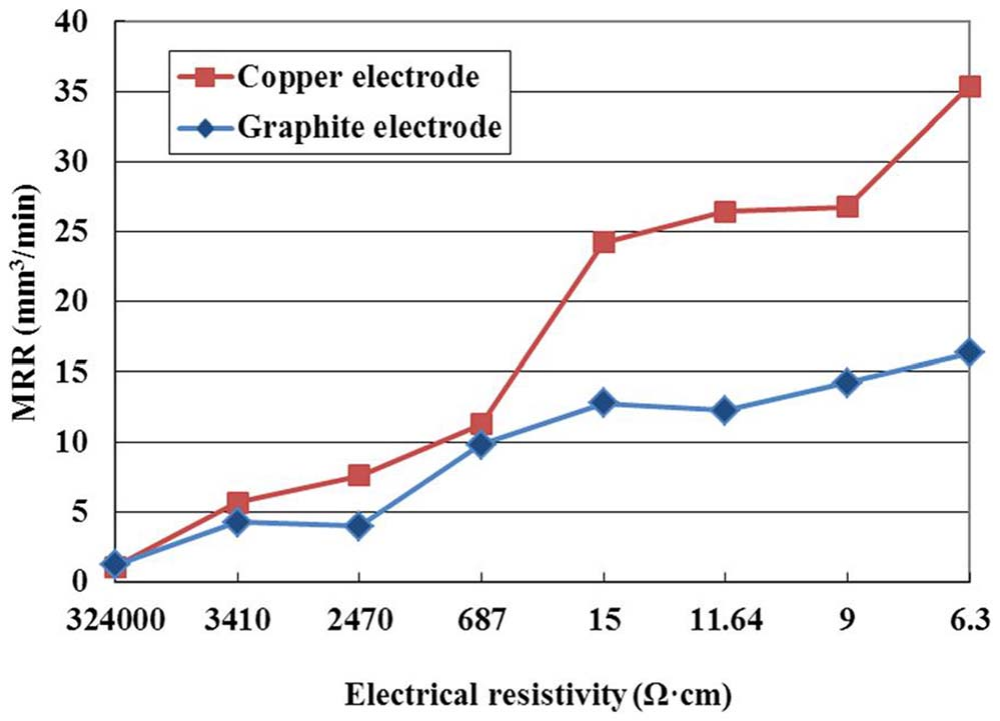
Effect of the electrode material on MRR.

**Figure 12:**
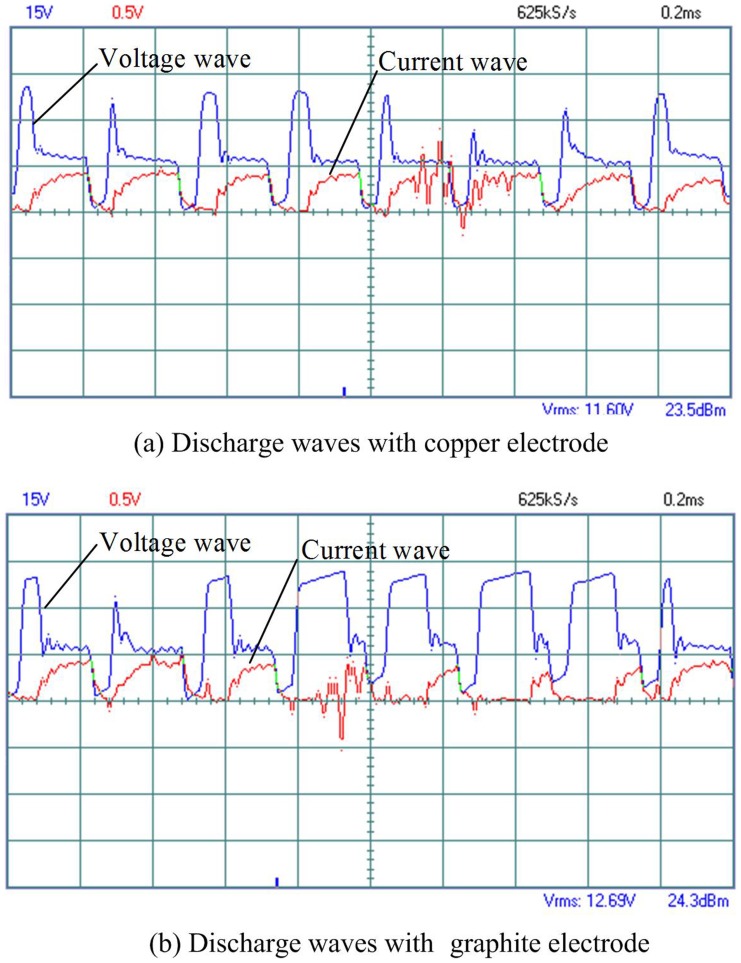
Discharge waves of the ZnO/Al_2_O_3_ ceramics with electrical resistivity of 11.64 Ω·cm and different electrodes.

The effect of the electrode material on MRR is illustrated in Figure 11. As shown in this figure, the MRR with copper electrode is higher than that with graphite electrode at the same electrical resistivity, especially when the electrical resistivity is low. There are many reasons causing this phenomenon. Compared with graphite electrode, the electrical conductivity of copper electrode is better, the discharge channel is formed relatively easily, the discharge delay time is shorter, as illustrated in Figure 12, and the discharge energy is higher, which enhances the workpiece material removal. Furthermore, when the copper electrode is used and the electrical resistivity is lower than 687 Ω·cm, the discharge channel is formed easily, the pulse utilization ratio is high, the discharge is violent, so the MRR is high.

**Figure 13:**
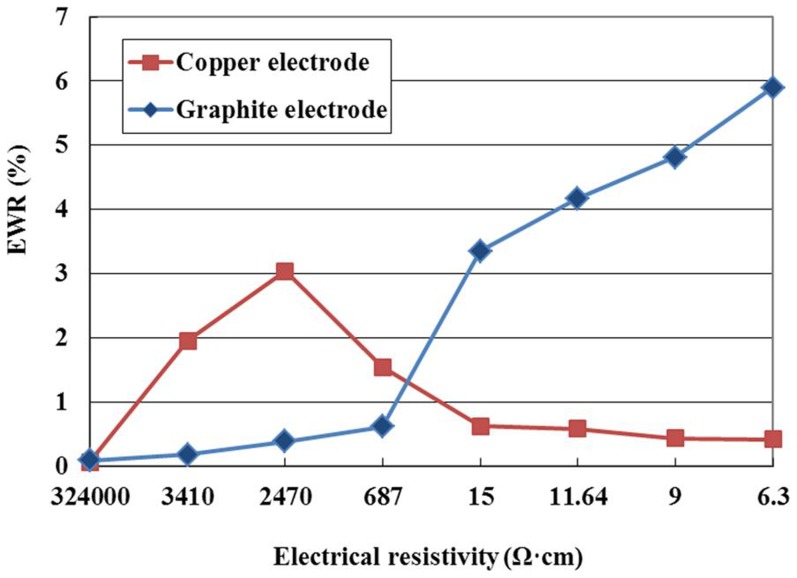
Effect of the electrode material on EWR.

Figure 13 shows the effect of the electrode material on EWR. It can be seen from this figure that the EWR with copper electrode initially increases with the decrease of electrical resistivity, and then decreases with the decrease of electrical resistivity, however, the EWR with graphite electrode initially increases slowly with the decrease of electrical resistivity, and then increases rapidly with the decrease of electrical resistivity. This is a complex phenomenon. When the electrical resistivity is high, the discharge channel occurs difficultly, and there are few discharges with any electrode material, so the electrode removal is low. When the copper electrode is used and the electrical resistivity is lower than 687 Ω·cm, the discharge is stable, a deposition layer can form on the electrode surface due to the decomposition of the dielectric and workpiece material attached to the tool electrode surface, and the electrode wear can be prevented by the protective effects of the deposition layer, so the EWR is low. However, when the graphite electrode is used and the electrical resistivity is lower than 687 Ω·cm, the discharge channel is formed easily, the discharge force is high, which can remove the graphite electrode easily due to its low hardness and soft texture; therefore, the EWR increases rapidly with the decrease of electrical resistivity.

It can also be seen from Figure 13 that the EWR with copper electrode is higher than that with graphite electrode when the electrical resistivity is high, and the opposite case happens when the electrical resistivity is low. This phenomenon can be explained as follows. When the electrical resistivity is high, the discharge channel occurs difficultly, compared with graphite electrode, the discharge with copper electrode occurs relatively easily, which enhances the electrode material removal, so the EWR with copper electrode is higher than that with graphite electrode. However, when the electrical resistivity is lower than 687 Ω·cm, the discharge channel is formed easily, the copper electrode wear can be prevented by the protective effects of the deposition layer, and the graphite electrode removal is enhanced due to its low hardness and soft texture, so the EWR with graphite electrode is higher than that with copper electrode.

**Figure 14:**
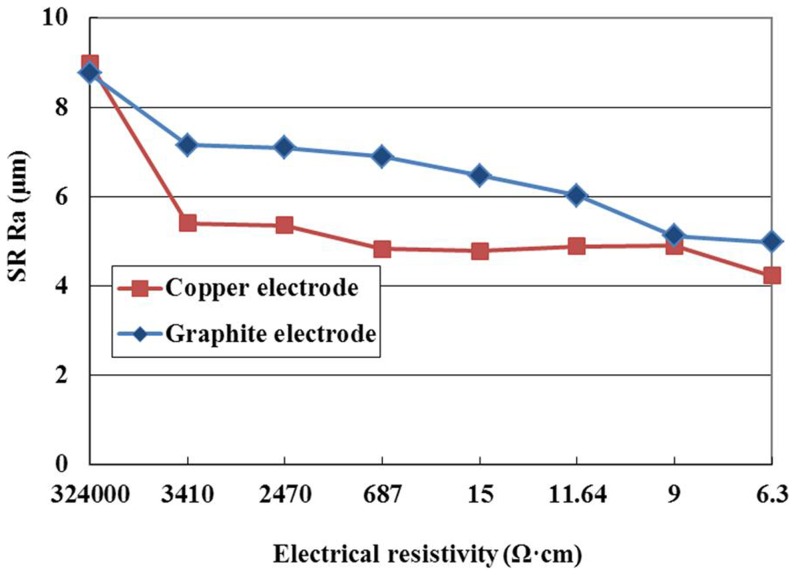
Effect of the electrode material on SR.

SR with different machining conditions is given in Figure 14. The figure indicates that the SR with copper electrode is lower than that with graphite electrode at the same electrical resistivity. Many things can cause this phenomenon. Compared with graphite electrode, the electrical conductivity of copper electrode is better, the discharge channel is formed relatively easily, and the workpiece is removed by vaporization and melting, which makes the workepiece surface smooth, so the SR with copper electrode is lower.

### Microstructure character analysis of ZnO/Al_2_O_3_ ceramic surface machined by EDM

#### Micro-topography of the machined surface

**Figure 15:**
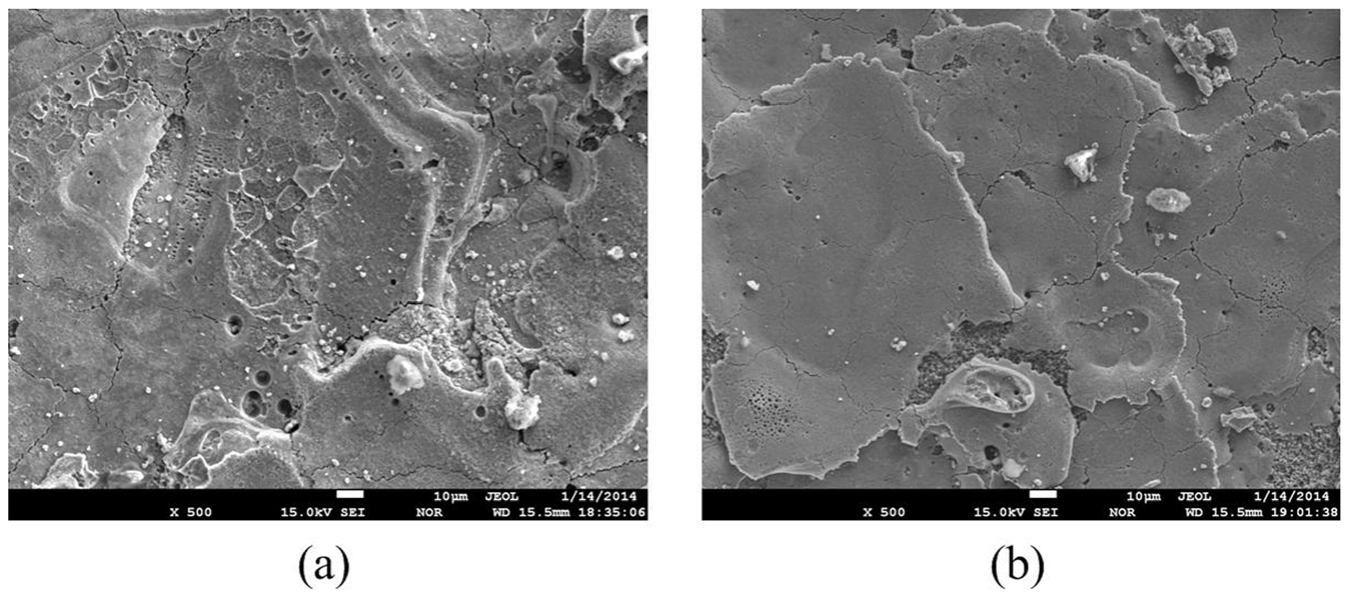
Micrographs of machined ZnO/Al_2_O_3_ ceramic surfaces with different tool polarities: (a) tool(-); (b) tool(+).

The micrographs of the machined ZnO/Al_2_O_3_ ceramic surface for pulse interval of 20µs and the electrical resistivity of 2470 Ω·cm with different tool polarities are illustrated in Figure 15. Many craters, droplets and micropores exist in the micrographs. The phenomenon indicates that the ZnO/Al_2_O_3_ ceramic is molten or evaporated by the sparking thermal energy. The formation of the craters on these surfaces is due to sparks that form on the surface generating melting or possible evaporation. As the spark ceases, most molten material is flushed away, but not all of the molten material can be removed because of the surface tension, and bonding forces between liquid and solid. The molten material remained on the workpiece surface is cooled by the working fluid, and some droplets are formed. The formation of micropores is ascribed to the ejection of gases that escape from the solidified material.

It is obvious that the surface is rougher, and the craters are bigger and deeper in case of negative tool polarity, when compared to positive tool polarity under the same conditions. As mentioned above, the bombardment effect by the electrons is stronger than that by positive ions during electrical discharge machining ZnO/Al_2_O_3_ ceramic. Stronger electrons bombardment results in bigger and deeper craters, leading to a rougher surface, as illustrated in Figure 15(a). Weaker positive ions bombardment results that more molten material re-solidified on the surface in case of positive tool polarity, leading to a smoother surface, as illustrated in Figure 15(b). This observation is in accordance with the roughness values, presented in Figure 7.

#### Micro-cracks on the machined surface

**Figure 16:**
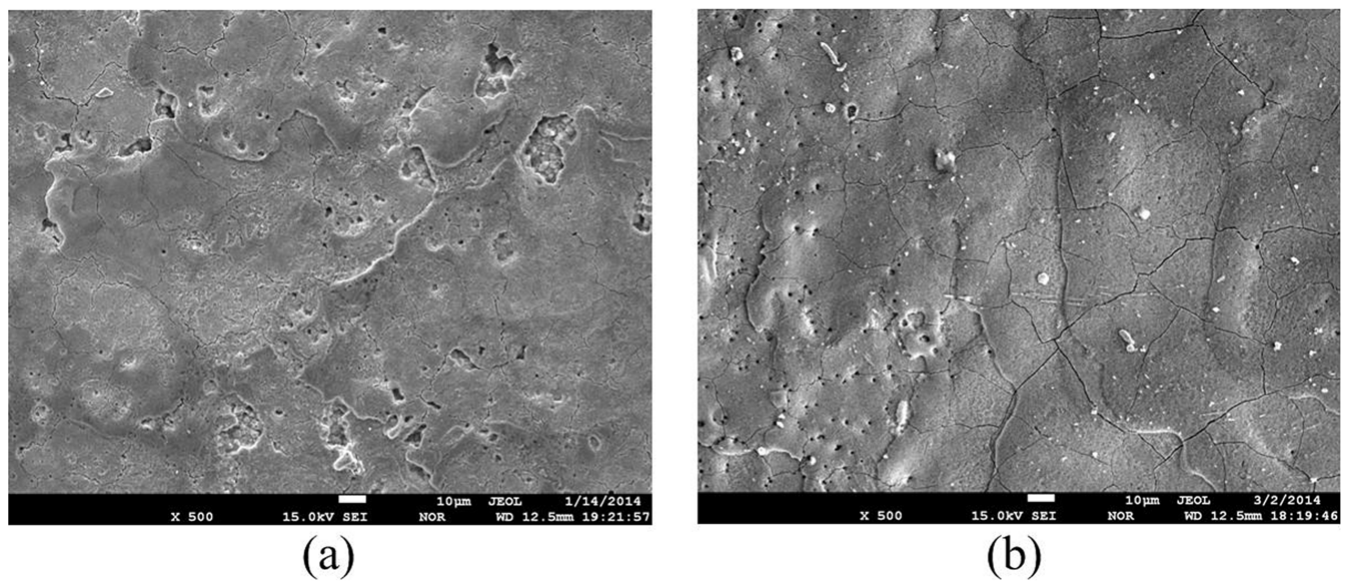
Microcracks on machined ZnO/Al_2_O_3_ surfaces with different electrical resistivities: (a) 15 Ω·cm; (b) 6.3 Ω·cm.

Figure 16 shows the micro-cracks on the machined ZnO/Al_2_O_3_ ceramic surfaces with different electrical resistivities. The microcracks formation is associated with the development of high thermal stresses during machining, and it is responsible for the thermal spalling removal of the ZnO/Al_2_O_3_ ceramic. It can also be seen from Figure 16 that the number and size of the micro-cracks on the machined surface increase with the decrease of electrical resistivity. The phenomenon can be explained as follows. As the electrical resistivity decreases, the discharge channel is formed relatively easily, the discharge becomes violent, the thermal impact and thermal stress increase, so the number and size of the micro-cracks on the machined surface increase with the decrease of electrical resistivity.

#### Compositions of the machined surface

**Figure 17:**
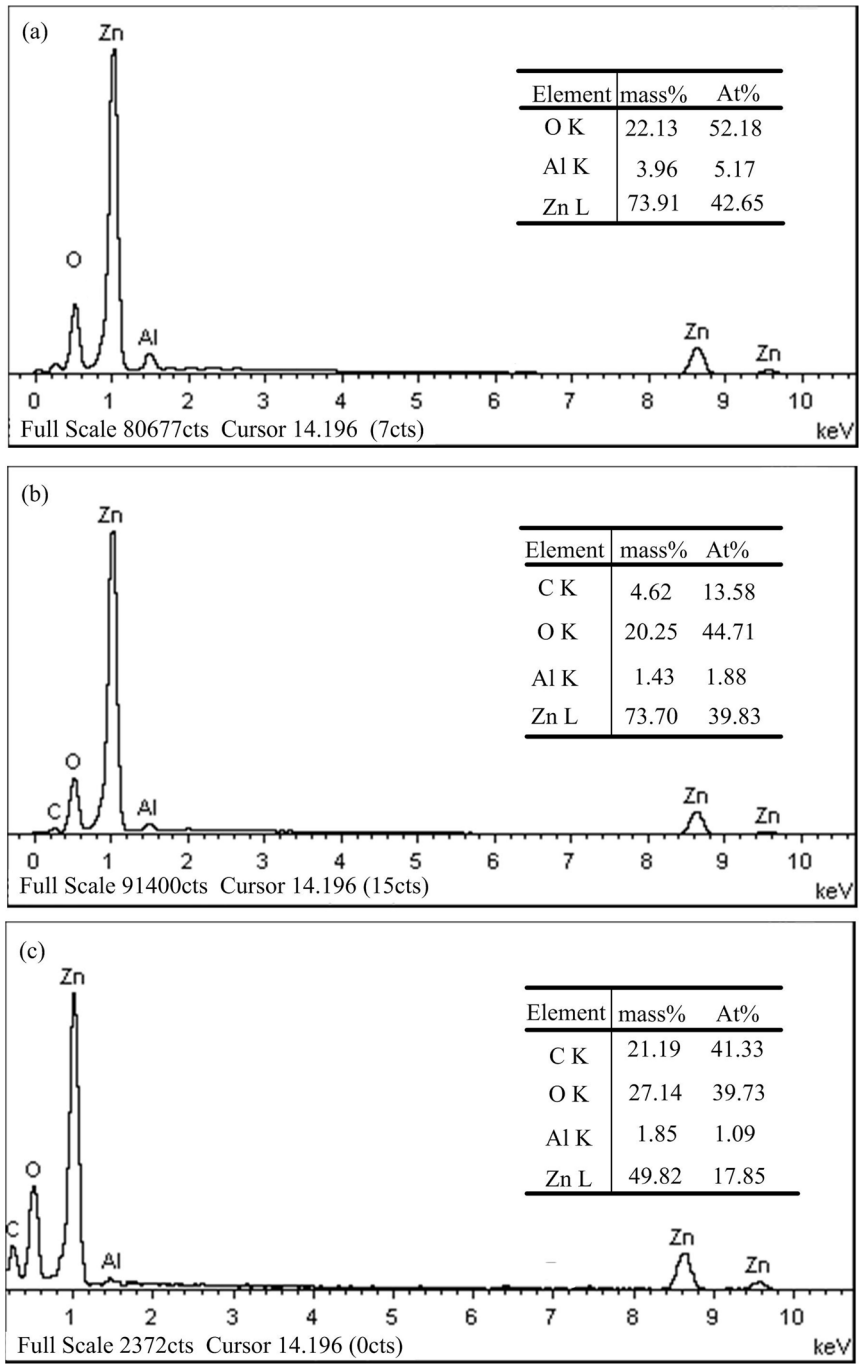
EDS analysis of ZnO/Al_2_O_3_ surfaces with different electrode materials: (a) unprocessed; (b) copper; (c) graphite.

Energy dispersive spectrometer (EDS) spectrum analysis is used to identify the elemental composition on the workpiece surface generated in different machining conditions. Figure 17 shows the EDS spectrum analysis of the ZnO/Al_2_O_3_ ceramic surface with the electrical resistivity of 6.3 Ω·cm and different electrode materials, in which Figure 17(a) shows the EDS spectrum analysis of the unprocessed ZnO/Al_2_O_3_ ceramic surface, Figure 17(b) shows the EDS spectrum analysis of the machined ZnO/Al_2_O_3_ ceramic surface with the copper electrode, and Figure 17(c) shows the EDS spectrum analysis of the machined ZnO/Al_2_O_3_ ceramic surface with the graphite electrode. It can be seen from Figure 17 that the prominent elements on the machined surface are carbon (C), oxygen (O), aluminum (Al), and zinc (Zn), whereas the prominent elements on the unprocessed surface are oxygen (O), aluminum (Al), and zinc (Zn). Moreover, the carbon percentage on the machined ZnO/Al_2_O_3_ ceramic surface is higher with the graphite electrode than that with the copper electrode. This is a complex phenomenon. During electrical discharge machining ZnO/Al_2_O_3_ ceramic, the working fluid can be decomposed, generating carbon element, which will deposit on the workpiece surface, so carbon element is detected on the machined surface. Moreover, compared with the copper electrode, more carbon will be produced with the graphite electrode due to its low hardness and soft texture, so the carbon percentage on the machined ZnO/Al_2_O_3_ ceramic surface is higher with the graphite electrode than that with the copper electrode.

## Conclusions

This study attempts to investigate the effects of the electrical resistivity and the EDM parameters on EDM performance of ZnO/Al_2_O_3_ ceramics. The following conclusions could be drawn from this study:

(1) The electrical resistivity of the ZnO/Al_2_O_3_ ceramic, which can be effectively machined by EDM, is high with the copper electrode, the negative tool polarity, and the shorter pulse interval. Moreover, the ZnO/Al_2_O_3_ ceramic with the electrical resistivity up to 3410 Ω·cm can be effectively machined by EDM with the appropriate machining condition.

(2) The EDM performance of ZnO/Al_2_O_3_ ceramic with the electrical resistivity higher than 687 Ω·cm, is obviously different from that with the electrical resistivity lower than 687 Ω·cm, when the electrode material changes.

(3) Under the same machining condition, the MRR increases with the decrease of electrical resistivity. At the same electrical resistivity, the higher MRR can be obtained with the negative tool polarity, the copper electrode, and the shorter pulse interval.

(4) The tool polarity, and pulse interval affect the EWR, respectively, and the lower EWR can be obtained with the negative polarity, the shorter pulse interval at the same electrical resistivity. Moreover, the electrical resistivity and electrode material have a combined effect on the EWR.

(5) Under most machining conditions, the SR decreases with the decrease of electrical resistivity. At the same electrical resistivity, the lower SR can be obtained with positive tool polarity, the longer pulse interval, and copper electrode.

(6) The machined surfaces are characterized by craters, droplets, micropores and microcracks. The removal mechanism during electrical discharge machining ZnO/Al_2_O_3_ ceramic consists of melting, evaporation and thermal spalling. The chemical compositions of the machined surface differ from the unprocessed surface due to the carbon element deposition from the working fluid and graphite electrode.
